# A Cross Structured Light Sensor and Stripe Segmentation Method for Visual Tracking of a Wall Climbing Robot

**DOI:** 10.3390/s150613725

**Published:** 2015-06-11

**Authors:** Liguo Zhang, Jianguo Sun, Guisheng Yin, Jing Zhao, Qilong Han

**Affiliations:** College of Computer Science and Technology, Harbin Engineering University, Harbin 150001, China; E-Mails: zhangliguo@hrbeu.edu.cn (L.Z.); yinguisheng@hrbeu.edu.cn (G.Y.); zhaojing@hrbeu.edu.cn (J.Z.); hanqilong@hrbeu.edu.cn (Q.H.)

**Keywords:** structured light sensor, laser stripe segmentation, weld line tracking, wall climbing robot

## Abstract

In non-destructive testing (NDT) of metal welds, weld line tracking is usually performed outdoors, where the structured light sources are always disturbed by various noises, such as sunlight, shadows, and reflections from the weld line surface. In this paper, we design a cross structured light (CSL) to detect the weld line and propose a robust laser stripe segmentation algorithm to overcome the noises in structured light images. An adaptive monochromatic space is applied to preprocess the image with ambient noises. In the monochromatic image, the laser stripe obtained is recovered as a multichannel signal by minimum entropy deconvolution. Lastly, the stripe centre points are extracted from the image. In experiments, the CSL sensor and the proposed algorithm are applied to guide a wall climbing robot inspecting the weld line of a wind power tower. The experimental results show that the CSL sensor can capture the 3D information of the welds with high accuracy, and the proposed algorithm contributes to the weld line inspection and the robot navigation.

## 1. Introduction

NDT is very important to guarantee the safe operation of lots of industry facilities, such as wind turbine towers and oil storage tanks. In these industrial situations, automatic weld line tracking can navigate moving wall climbing robots and improve the testing performance significantly. From the perspective of sensors, structured light is a typical vision sensor, which possesses the merits of simplicity, noncontact, strong anti-interference abilities and others. It has been widely used in many fields, such as robot navigation [1], automatic welding [2], industry inspection [3,4], 3D measurement [5,6,7] and quality control [8,9]. Unlike weld seam detection in a welding process, weld line tracking for NDT is usually performed outdoors; therefore the sensor is challenged by noise and illumination intensity variations.

The structured light actually has two subclasses: multi-pattern (coded-pattern) and fixed-pattern. The multi-pattern approaches are accurate in the controlled environment of stationary scenes due to their high signal-to-noise ratio (SNR), whereas fixed-pattern methods are robust to ambient lighting changes, but they have less accuracy [10]. Generally, methods for 3D measurement and reconstruction of an object use coded-pattern structured light with high precision and fast speed. However, these methods need to be operated in relatively ideal scenes (indoors). In outdoor scenes, the coded-pattern may fail due to ambient noises and reflections from the object surface [11,12,13]. Thus, the methods based on fixed-pattern (that is, typical liner structured light) are more appropriate for vision navigation of robots in outdoor scenes. The structured light with single laser stripe is the most widely-used approach in industrial environments. For instance, Usamentiaga and Molleda *et al.* [14] used single laser stripes to inspect and control the flatness of steel strips in real-time. For removing the effects of vibrations, two and multiple laser stripes are also used in uncontrolled environments [5,15].

When using a structured light sensor, robust stripe segmentation is a decisive step that determines the accuracy of tracking and localization of the weld line to be considered, because it describes the local 3D details of the weld line surface. Most research focuses on the peak detection which is used to construct the skeleton of the laser stripe afterwards. The most common methods are the search for the maximum intensity or the detection of the center of the stripe in the image with the use of Linear Approximation, Center of Mass, Gaussian Approximation, Parabolic Estimator, Blais and Rioux Detector. Fisher *et al.* [16] gave a comparison of several most common approaches ranging from simple methods to complex ones and concluded that all of them displayed performance within the same range. Haug indicated that the center of mass method produces the best results [17]. Strobl *et al.* [18] presented a stripe segmentation algorithm also based on the center of mass. They detected the stripe edge by using the Sobel operator, then modified the edge points by means of a color look-up table and width limits, both of which need a clean background. Li *et al.* proposed to use temporal data association to filter out the illumination noise from arc lights or splashes in video sequences [19]. However, it is difficult to extend this approach to outdoor scenes because the illumination variation in outdoor scenes is quite different from arc lights or splashes. Molleda *et al.* proposed a valid method based on the center of mass [20,21]. They use interferential filters to acquire parts of the image which match the laser wavelength and this reduces greatly noises. The laser stripe is segmented with the use of an improved Split-and-Merge approach from the central points. In their paper, several different approximation functions (linear, quadratic, and Akima splines) are evaluated. Ofner *et al.* used a line walking algorithm to merge the line gaps, which segment the stripe by looking for maximum values which are higher than a certain threshold in the adjacent row [22]. Forest *et al.* used a FIR filter approach to detect the peak position of the laser stripe under different noise levels (at different signal-to-noise ratios) [23]. However, the problem of long*-*duration variation of illumination intensity has remained a challenge to segment stripe approaches.

Most peak detection approaches assume that the distribution of the laser illumination approximates a Gaussian distribution. In these researches, researchers segment laser stripe only under low-level noise conditions according to local information. Thus these approaches are sensitive to the environmental illumination and can only be applied to scenes with high contrast between the laser illumination and background. Because of the above demerits, these methods cannot be directly applied in outdoor and industrial environments with strong illumination and high noise.

Some approaches segment the laser stripe according to global information. In [24], based on the snake model, the spring model improves the segmentation results and shows insensitivity to local noise. The method is robust to the low-quality images caused by laser speckle and illumination variations along the laser line. It is worth noting that it obtains an accurate, complete, robust and smooth centerline of the stripe. In many other studies, most of detection methods concentrate on the detection accuracy of the stripe, often at the expense of robustness and effectiveness.

In this paper, we design a cross-structure light sensor for weld line detection and tracking of a wall climbing robot working in outdoor environments. The laser stripe projected by CSL can reflect the height of weld convexity and detect horizontal and vertical weld lines simultaneously. When the robot moves along a weld line, only a stripe is used to detect the weld line. As the robot is close to a T-intersection of two weld lines, two convex arcs will appear at the strips. The sensor can simultaneously detect two weld lines, which are used to plan the motion path of the robot (make a turn). The problem of the stripe segmentation and centre points localization is formulated through three steps: firstly, to eliminate the effect of illumination, the best monochromatic value space is calculated by the minimum entropy model. Secondly, the minimum entropy deconvolution model is used to acquire the enhancement of the laser stripe. Thirdly, based on the results of the two steps above, all centre points of the stripe are localized by Steger’s method [25]. The proposed algorithm segments the stripe according to global information and overcomes gaps and strong noises.

In the experimental results, the stripe segmentation method is used in a CSL sensor of wall climbing robot. We quantitatively compare the proposed approach with four other approaches to verify the performance of the segmenting stripe. Besides, 3D measurement results of the weld line and path tracing of the wall climbing robot are shown. The controller of the robot is described in [26] and the weld line localization is described in [27], so the details of both are omitted in this paper.

The remainder of this paper is organized as follows: in Section 2, a wall climbing robot and CSL sensor are introduced. In Section 3, the approaches of segmenting the laser stripe and localizing centre points are presented. In Section 4, the experimental results and discussion are presented. The conclusions of the paper are given in Section 5.

## 2. Cross Structured Light Sensor

### 2.1. The Robot Platform

**Figure 1:**
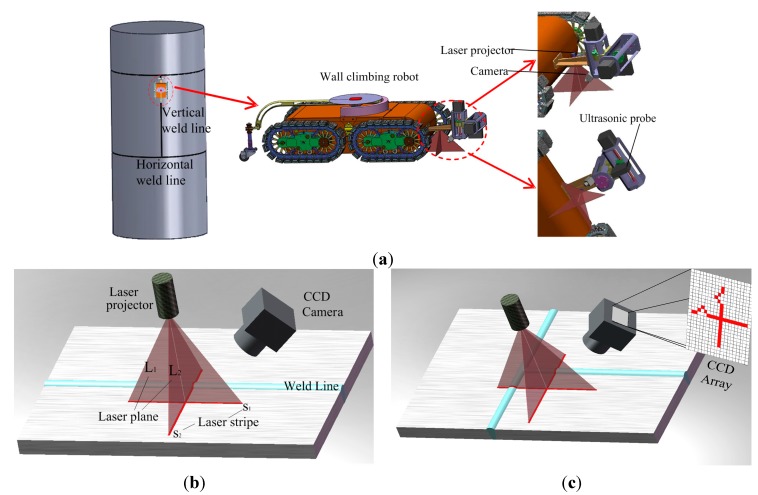
Illustration of the weld line inspection system composed of a wall climbing robot, an NDT device and a CSL sensor. (**a**) The system; (**b**) Detecting a straight weld line; and (**c**) T-intersection of weld lines.

The quality inspection system of the weld line is shown in Figure 1a. It is composed of a wall climbing robot, an ultrasonic NDT device and a CSL sensor. Figure 1b,c illustrate the laser projector and image capturing CCD on a straight weld line and cross weld lines, respectively. Two cross laser planes are projected from the laser projector, forming a convex light stripe around the weld line of the welding surface. The laser projector is fixed on the robot and perpendicular to the welding. Two stripes *s*_1_ and *s*_2_ are formed by the intersection lines between the weld and two orthogonal laser planes *L*_1_ and *L*_2_. According to the triangulation measurement method, the 3D information of the weld surface can be calculated by transforming the points of the stripes from the image coordinate system to the global coordinate system.

In general, when the robot moves along the straight weld line, only a convex arc exists on the stripe *s*_2_, as shown in Figure 1b. When the robot is close to a T-intersection of vertical and horizontal weld lines, two convex arcs will appear at the strips, as shown in Figure 1c. The sensor can simultaneously detect the locations of the horizontal and the vertical weld lines, which are used to plan the motion path of the robot.

### 2.2. Model of CSL Sensor

**Figure 2:**
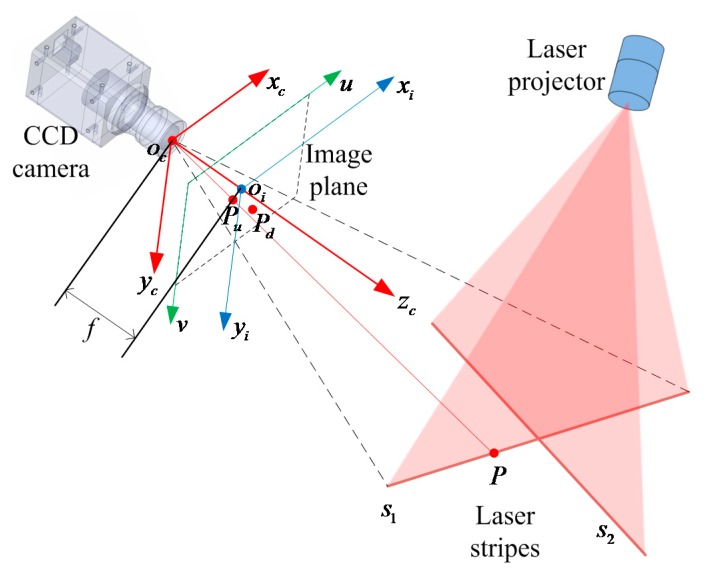
Model of the CSL sensor.

According to the above principle, Figure 2 describes the model of the CSL sensor. *o_c_*-*x_c_y_c_z_c_* denotes the camera coordinate system. *o_c_o_i_ = f* denotes the focal length of the camera. *o_i_*-*x_i_y_i_* denotes the image coordinate system. In camera coordinate system, two mutually orthogonal laser planes *L*_1_ and *L*_2_ are defined as:

(1) $$\left\{ \begin{array}{l} a_{1}x_{\text{c}}+b_{1}y_{c}+c_{1}z_{c}+1=0\\ a_{2}x_{c}+b_{2}y_{c}+c_{2}z_{c}+1=0 \end{array}\right.$$

where *a*_1_*a*_2_ + *b*_1_*b*_2_ + *c*_1_*c*_2_ = 0.

There is an arbitrary point *P* on the stripe *s*_1_. *P_c_* = [*x_cp_*, *y_cp_*, *z_cp_*,]*^T^* denotes the coordinate of point *P* in camera coordinate system and *P_u_* = [*x_up_*, *y_up_*]*^T^* denotes the coordinate of point *P* in the image coordinate system. The pixel location of *p_u_* is represented by [*u_up_*, *v_up_*]*^T^*. Assuming the width and height of a pixel are *dx*(mm) and *dy*(mm), respectively, in the image coordinate system the transformational relation from millimeter coordinate to pixel coordinate is described as:

(2) $$\left[\begin{array}{c} u_{up}\\ v_{up} \end{array}\right]=\left[\begin{array}{cc} 1/dx & 0\\ 0 & 1/dy \end{array}\right]\left[\begin{array}{c} x_{up}\\ y_{up} \end{array}\right]+\left[\begin{array}{c} u_{0}\\ v_{0} \end{array}\right]$$

where, the point [*u*_0_, *v*_0_]*^T^* is the intersection coordinates of the optical axis and the image plane, *i.e.*, the principal point of the camera in the image plane. According to the pinhole model, the transformational relation from image coordinate to camera coordinate is described as:

(3) $$\left[\begin{array}{c} u_{up}\\ v_{up}\\ 1 \end{array}\right]=\left[\begin{array}{ccc} k_{x} & 0 & u_{0}\\ 0 & k_{y} & v_{0}\\ 0 & 0 & 1 \end{array}\right]\left[\begin{array}{c} x_{cp}/z_{cp}\\ y_{cp}/z_{cp}\\ 1 \end{array}\right]=K\left[\begin{array}{c} x_{cp}/z_{cp}\\ y_{cp}/z_{cp}\\ 1 \end{array}\right]$$

where $k_{x}=\frac{f}{dx}$, $k_{y}=\frac{f}{dy}$; *K* is the camera’s intrinsic matrix.

Actual lenses do not obey the ideal pinhole model due to lens distortion [28]. When modeling the camera, correction of radial distortions and tangential distortions is often used to improve the measurement precision [29,30]. In Figure 2 point *p_u_* is the unobservable distortion free image coordinates, and *p_d_* = [*x_d_*, *y_d_*]*^T^* is the corresponding coordinates with distortion correction. *p_u_* and *p_d_* are decided by the positions of the points in the image plane and described by:

(4) $$\{\begin{array}{c} x_{up}=k_{1}x_{d}\left(x_{d}^{2}+y_{d}^{2}\right)+k_{2}x_{d}{\left(x_{d}^{2}+y_{d}^{2}\right)}^{2}+p_{1}\left(2x_{d}^{2}+{\left(x_{d}^{2}+y_{d}^{2}\right)}^{2}\right)+p_{2}\left(2x_{d}y_{d}\right)\\ y_{up}=k_{1}y_{d}\left(x_{d}^{2}+y_{d}^{2}\right)+k_{2}y_{d}{\left(x_{d}^{2}+y_{d}^{2}\right)}^{2}+p_{2}\left(2y_{d}^{2}+{\left(x_{d}^{2}+y_{d}^{2}\right)}^{2}\right)+p_{1}\left(2x_{d}y_{d}\right) \end{array}$$

where parameters (*k*_1_, *k*_2_) of radial distortion and parameters (*p*_1_, *p*_2_) of tangential distortion are used to model the camera. According to Equations (1)–(4), we can calculate the parameters of the camera model and the coordinates of points on the laser stripe.

### 2.3. Calibration of CSL Sensor

In the sensor calibration process, the intrinsic parameters of the camera and laser planes parameters need to be computed. Table 1 lists these corresponding parameters and their physical meanings. Where *o_r_*-*x_r_y_r_z_r_* denotes the robot coordinate system.

**Table 1:** Parameters of the CSL sensor.

Category	Parameters	Physical Meaning
Camera intrinsic parameters	(*f_x_*, *f_y_*)	Focal length in the *x*, *y* direction
	(*u*_0_, *v*_0_)	Principle point coordinates
	(*k*_1_, *k*_2_)	Radial distortion parameters
	(*p*_1_, *p*_2_)	Tangential distortion parameters
Light plane equations	(*a*_1_, *b*_1_, *c*_1_)	Laser plane *L*_1_ equation coefficients
	(*a*_2_, *b*_2_, *c*_2_)	Laser plane *L*_2_ equation coefficients
	∠ *l*_1_*ol*_2_	Angle between *L*_1_ and *L*_2_
Global parameters	*R_cr_*	Rotation from *o_c_*-*x_c_y_c_z_c_* to *o_r_*-*x_r_y_r_z_r_*
	*T_cr_*	Translation from *o_c_*-*x_c_y_c_z_c_* to *o_r_*-*x_r_y_r_z_r_*

**Figure 3:**
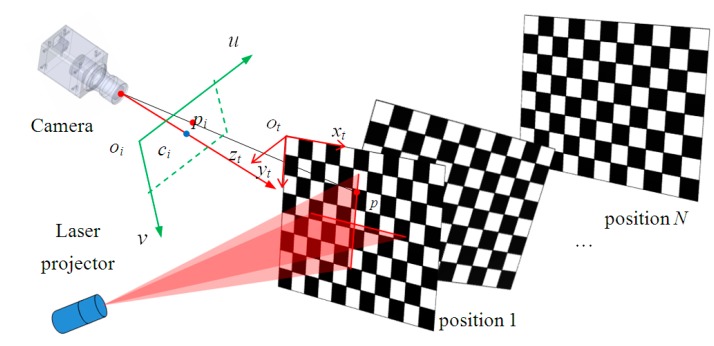
Sensor calibration.

The camera and the planar checkerboard target can move freely. The relative camera positions of the checkerboard among different poses are randomly located, as shown in Figure 3. At each position, the camera respectively captures an image with laser stripe and an image without laser stripe by on-off control of the laser projector. The image with laser stripe is used to calculate the equations of the laser planes, and the image without laser stripe is used to calibrate the intrinsic parameters. A known target and Zhang’s method [31] are used to determine the intrinsic parameters of the camera, in which a camera observes a planar checkerboard target from different perspectives. The calibration procedure gives a closed form solution, followed by a nonlinear refinement based on the maximum likelihood criterion.

In Figure 3 the planar checkerboard target is also used to calibrate the laser planes. *o_t_*-*x_t_y_t_z_t_* is a target coordinate system of the target plane and it is used as the global coordinate system during calibration. Under this condition, the *z* coordinates of all points of the target plane equals zero. From a 3D target plane coordinate system to 2D image coordinate system, the translation is given as:

(5) $$s\tilde{m}=A\left[\begin{array}{cccc} r_{1} & r_{2} & r_{3} & t \end{array}\right]{\left[\begin{array}{cccc} x_{t} & y_{t} & z_{t} & 1 \end{array}\right]}^{T}=A\left[\begin{array}{cc} R & t \end{array}\right]\tilde{M}$$

where $\tilde{m}={\left[\begin{array}{ccc} u & v & 1 \end{array}\right]}^{T}$ are the homogeneous coordinates of $m={\left[\begin{array}{cc} u & v \end{array}\right]}^{T}$; $\tilde{M}={\left[\begin{array}{ccc} x_{t} & y_{t} & \begin{array}{cc} z_{t} & 1 \end{array} \end{array}\right]}^{T}$ are the homogeneous coordinates of $M={\left[\begin{array}{cc} x_{t} & \begin{array}{cc} y_{t} & z_{t} \end{array} \end{array}\right]}^{T}$ in the 2D target plane; *s* is an arbitrary scale factor; 3 × 3 matrix $R=\left(\begin{array}{ccc} r_{1} & r_{2} & r_{3} \end{array}\right)$ and 3 × 1 matrix $t={\left(\begin{array}{ccc} t_{1} & t_{2} & t_{3} \end{array}\right)}^{T}$ are the rotation matrix and translation vector between two coordinate systems, respectively. According to $z_{t}=0$ on the target plane, the translation between $\tilde{m}$ and $\tilde{M}$ is described as:

(6) $$\tilde{m}=\frac{1}{s}A\left[\begin{array}{ccc} r_{1} & r_{2} & t \end{array}\right]{\tilde{M}}^{\prime }=H{\tilde{M}}^{\prime }$$

where the homography matrix *H* is given as $H={\left[\begin{array}{ccc} h_{1} & h_{2} & h_{3} \end{array}\right]}^{T}$ ($h_{i}$, *i* = 1, 2 or 3 is row vector of *H*); ${\tilde{M}}^{\prime }={\left[\begin{array}{ccc} x_{t} & y_{t} & 1 \end{array}\right]}^{T}$. *H* can be coarsely calculated with Equation (5) with at least four non-collinear points. Then a maximum likelihood estimation method is used to calculate the accurate *H* by minimizing the objective function:

(7) $$F=\min\sum_{i}{\left\|m_{i}-\frac{1}{h_{3}^{T}M_{i}}\left[\begin{array}{l} h_{1}^{T}M_{i}\\ h_{2}^{T}M_{i} \end{array}\right]\right\|}^{2}$$

where $m_{i}={\left(\begin{array}{ccc} u_{i} & v_{i} & 1 \end{array}\right)}^{T}$ and $M_{i}={\left(\begin{array}{ccc} x_{i} & y_{i} & 1 \end{array}\right)}^{T}$ are the homogeneous coordinates of the *i*th feature point in image coordinate system and its corresponding point in target plane coordinate system, respectively. The minimizing problem can be solved by the Levenberg-Marquardt algorithm [32]. The initial value can be obtained from a linear least squares method.

**Figure 4:**
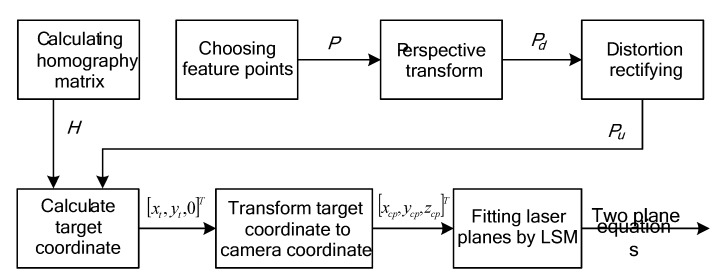
Flowchart of the feature point coordinate calculation.

**Figure 5:**
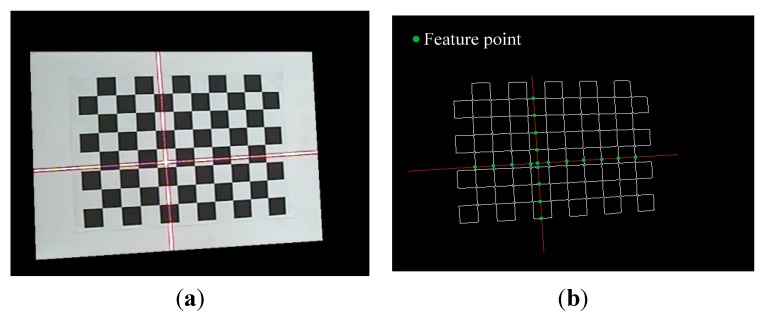
Image used for calibration of laser planes. (**a**) Capturing image with laser stripes; and (**b**) extracting feature points on the stripes.

After the cameral calibration, we follow the flowchart shown in Figure 4 to calculate the equations of the laser planes. On the checkerboard, the intersection points of the edges of black/white squares and the stripes are defined as feature points, as shown in Figure 5. The checkerboard is placed at different positions to get the camera coordinates of sufficient points on the stripes (Figure 3). The space equations of laser planes *L*_1_ and *L*_2_ are fitted using a least square method (*LSM*).

## 3. Laser Stripe Segmentation and Centre Points Localization

Outdoors the noise caused by sunlight has a distinguishing characteristic: the values of the R, G and B components are nearly equal. Sometimes the needed information of the image may not show clearly in RGB color space. Therefore, transforming the color space to a monochromatic value space may be helpful to highlight the characteristics of the laser and conveniently segment the stripe. In the paper, a linear transformation is used to obtain a monochromatic value image from RGB images.

### 3.1. Preprocessing Based on Monochromatic Value Space

In a discrete color image *c_ij_* (with a size of M × N), color values of a pixel are given as three corresponding tristimulus *R_ij_*, *G_ij_*, and *B_ij_*. The linear transformation is defined as Equation (8):

(8) $$I_{ij}=\omega_{r}R_{ij}+\omega_{g}G_{ij}+\omega_{b}B_{ij}$$

In the Equation (8), *I_ij_* is the desired monochromatic value image, and i∈[1,2,...,M], j∈[1,2,...,N], ω*_r_*, ω*_g_*, and ω*_b_* ∈ *R*. In order to segment the stripe the characteristics of laser should be take into consideration. An objective function needs defining to search the optimal ω*_r_*, ω*_g_*, and ω*_b_*.

**Figure 6:**
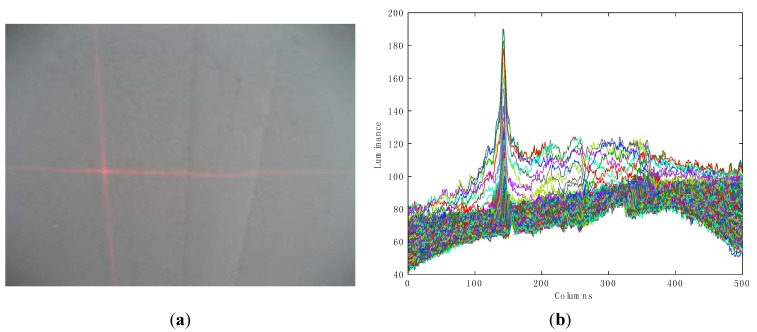
Example of laser profiles. (**a**) Captured image including the cross lasers stripe on the weld line; (**b**) Superposition of the luminance values row by row.

Owing to the concentration and the strong brightness of the laser beam, one of the remarkable characteristics of laser is the high concentration of energy, which makes the stripe show a waveform with a few spikes (as shown in Figure 6b, a laser profile example, which is obtained through showing all the image row vectors). It means that the contrast between the laser stripe and background is high. The objective function should retain and enhance the feature so that it will become much easier to segment the laser stripe after transformation. Thus, the contrast can be used to construct the objective function. In Figure 6a, intensity values of the pixels within the laser stripe are close to their average. In these areas, the greater the energy concentration is, the higher the contrast will be and the higher the Kurtosis will be. Therefore, it is reasonable to define the contrast as kurtosis:

(9) $$K=\frac{\kappa_{4}}{\kappa_{2}^{2}}=\frac{\mu_{4}}{\sigma^{4}}-3$$

In the Equation (9), kurtosis *K* is defined as the ratio of fourth-order cumulant (*FOC*) *κ*_4_ to square of the second-order cumulant (*SOC*) $\kappa_{2}^{2}$. $\mu_{4}$ and σ are respectively the fourth central moment and the standard deviation of the energy distribution of the laser.

The transformation result of the stripe is expected to be an impulse-like signal which is orderly and furnished with high kurtosis. The background is of great disorder and low kurtosis. For instance, in a communication system, disorder is equivalent to the concept of entropy. There is a positive correlation between the entropy and the random nature of information. For this reason, Wiggins [33] first presented the minimum entropy deconvolution technique. He proposed to maximize a norm function called the Varimax Norm, which is equivalent to maximized kurtosis with assumed zero-mean [34]. The transformation model can be named as minimum entropy model. When *K* < 0, the Equation (9) can be modified as the Equation (10):

(10) $$abs\left(K\right)=\left|\frac{\mu_{4}}{\sigma^{4}}-3\;\right|$$

Thereupon, a maximized function of Equation (10) which is differentiable everywhere can be defined as the square of kurtosis, as shown in Equation (11):

(11) $$\max\left(abs\left(K\right)\right)=\left|\frac{\mu_{4}}{\sigma^{4}}-3\right|\Leftrightarrow \max\left(K^{2}\right)={\left(\frac{\mu_{4}}{\sigma^{4}}-3\right)}^{2}$$

If the image is taken as a multi-channel signal (with *N* segments and *M* elements per segment), the Kurtosis can be written as:

(12) $$K^{2}={\left(\sum_{j=1}^{N}\frac{\sum_{i=1}^{M}{\left(I_{ij}-\mu_{j}\right)}^{4}}{{\left(\sum_{i=1}^{M}{\left(I_{ij}-\mu_{j}\right)}^{2}\right)}^{2}}-3\right)}^{2}$$

In the Equation (12), μ*_j_* is the mean of column *j* in the transform image *I_ij_*. To obtain the solution of the maximizing Equation (12), *K* and ω need to satisfy the Equation (13):

(13) $$\frac{\partial K}{\partial \omega_{r}}=0,\;\frac{\partial K}{\partial \omega_{g}}=0,\;\frac{\partial K}{\partial \omega_{b}}=0\;$$

Obviously, it is difficult to solve Equation (13), but the maximum value of *K* can be approximately calculated according to [35]. An infinite color feature space set is determined by the continuous coefficients in Equation (8). ω*_r_*, ω*_g_*, and ω*_b_* can be learned from training data by the maximum likelihood estimation or the maximum posteriori estimation. For the convenience of calculation, ω*_r_*, ω*_g_*, and ω*_b_* are discretized as integers, and their value range is limited in [−2, 2]. Then Equation (13) is solved by the exhaustion method. Considering that red laser light is used in the experiments, the *R* component of the captured image has higher intensity, so it is reasonable to define ω*_r_* ≥ 0, that is, ω*_r_* ∈ {0, 1, 2}, ω*_g_*, ω*_b_* ∈ {−2, −1, 0, 1, 2}, (ω*_r_*, ω*_g_*, ω*_b_*) ≠ (0, 0, 0). Then, the parameters can be solved by the traversal method.

**Figure 7:**
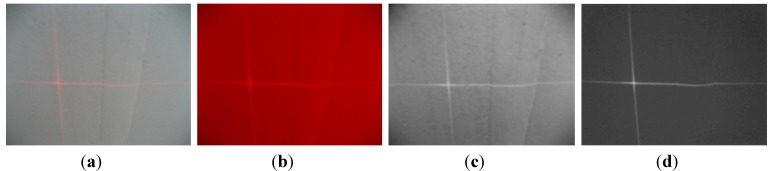
The color transport result: (**a**) captured image; (**b**) *R* component; (**c**) Grayscale;(**d**) the monochromatic value of *R-G.*

In Figure 7, the optimal coefficient vector of the monochromatic value image is (ω*_r_*, ω*_g_*, ω*_b_*) = (1, −1, 0), and the monochromatic value space is *R-G*. Compared with the color value *R* of the stripe and grayscale (*i.e.*, (ω*_r_*, ω*_g_*, ω*_b_*) = (0.30, 0.59, 0.11)) of the original image, *R-G* is more effective in suppressing noise and improving the SNR. On the one hand, it distinguishes the laser from the background more efficiently. On the other hand, it has much smaller amount of data (one-third of Figure 7a) than that of the original image.

### 3.2. Stripe Segmentation Based on Minimum Entropy Deconvolution(MED)

**Figure 8:**
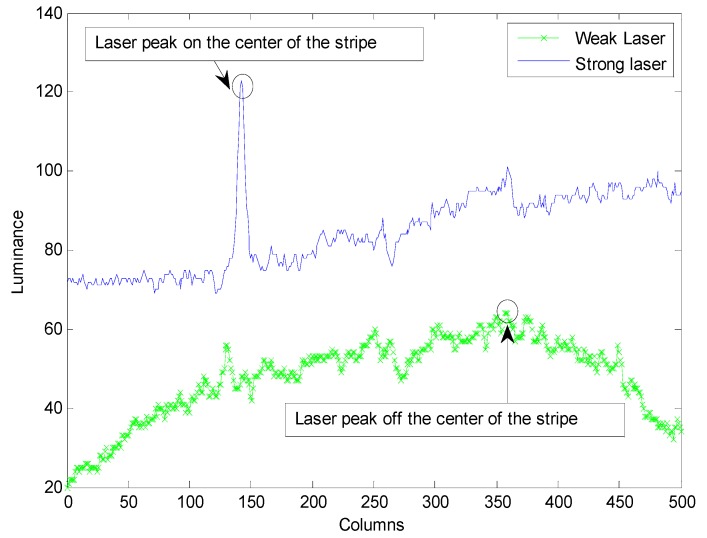
Laser peaks and their locations.

Ideally, the horizontal (and vertical) illumination distributions of stripes are independent, and their intensity conforms to a Gaussian distribution. When the laser projects onto the surface of an object, the distribution form will not change, but the stripe will deform with the change of the object surface geometrical shape. In an ideal situation, the laser peaks are in the center of the stripe composed of the intensity maximum along each column (or row). The laser stripe can be segmented only according to the peaks. However, in real environments, captured images contain various noises such as laser speckle, ambient noise, electrical noise, quantization noise, energy diffusion and excessive saturation [23]. Because of these noises, it is difficult to produce reliable stripe centers by simply calculating the maximum intensity along each profile of the laser stripe. The laser intensity does not always conform to Gaussian distribution and the maximum-valued locations of some columns (or rows) may not be in the center of the stripe. In Figure 8, it shows that some points off the center of stripe replace the stripe’s center points as the maximum points. From the prospect of signal processing, the structure information and the consistency of laser stripe signal are destroyed. Hence some actions should be taken to enhance the energy concentration of the laser stripe to return to their original locations.

**Figure 9:**
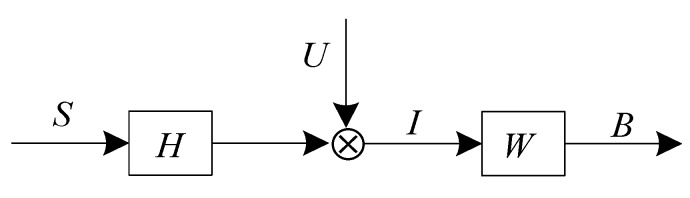
The model for 2D deconvolution.

After an appropriate monochromatic value space is chosen, to improve the image quality, the next step is to reset the peak points (theoretical maximum) back to their original positions. This process can be described as 2D image deconvolution. Figure 9 shows the algorithmic model [33,35,36].

It can be generally formulated as:

(14) I=U(H∗S)

(15) B=W∗I

Because the image is a set of the discrete points, Equations (14) and (15) are described as the following equations:

(16) $$I_{ij}=U\left(\sum_{k,l}H_{kl}S_{i-k+1,j-l+1}\right)$$

(17) $$B_{ij}=\sum_{k,l}W_{kl}I_{i-k+1,j-l+1}$$

In the Equations (14)–(17):

- *S* denotes the laser stripe;
- *H* denotes the point spread function of the optical imaging system;
- *U* denotes a noise function;
- *I* denotes the acquired image;
* denotes the 2D convolution operator;
- (*i*, *j*) is discrete spatial coordinates;
- *W* denotes the finite impulse response (FIR) filter, *W* = 0 if *i* < 1or *j* < 1, and W*H = δ*_i-_*_Δ*i*,*j-*Δ*j*_, where δ*_ij_* is the Krönecker delta (discrete impulse signal) [37], and Δ*i*, Δ*j* are the phase delay;
- *B* denotes the recovered image.

The goal of solving the deconvolution is to find the convolution kernel *W* by the maximized kurtosis *K*, so that *B_ij_ ≈* α*S_i-_*_Δ*i*,*j-*Δ*j*_, in which α is a scale factor. *MED* is an effective technique for deconvolving the impulsive sources from a mixture signals. In [38], an iterative deconvolution approach is proposed. A FIR filter is used to minimize the entropy of the filtered signal, *i.e.*, it searches for an optimum set of filter coefficients, which can recover the output signal with the maximum value of Kurtosis. This process will eventually enhance energy concentration and the structured information in the output signal [39,40,41], recovering an impulsive signal which will be more consistent than before.

Taking the input image as a multi-channel signal in columns (with *N* segments and *M* elements per segment) or in rows (with *M* segments and *N* elements per segment), in the former case, the horizontal component of the laser stripe is largely restored, and meanwhile, the vertical component information is suppressed, and *vice versa*. Then the whole recovered information of the laser stripe can be obtained by executing the two operations separately. The model in columns to extract the horizontal laser line can be formulated as:

(18) $$B_{ij}=\sum_{k=1}^{L}W_{k}I_{i-k+1,j},(k=1,...,L)$$

(19) $$O\left(W_{k}\right)={\left(\sum_{j=1}^{N}\frac{\sum_{i=1}^{M}{\left(B_{ij}-\mu_{Bj}\right)}^{4}}{{\left(\sum_{i=1}^{M}{\left(B_{ij}-\mu_{Bj}\right)}^{2}\right)}^{2}}-3\right)}^{2}$$

In Equations (18) and (19), μ*_Bj_* is the mean of column *j* of *B_ij_*, and *L* is the order of the filter, both of which have significant impact on the *MED* outputs. The form of objective function can be described by Equation (19).

The above model is similar to Wiggins’ method except for the objective function, and it will not influence the solution procedure. The *MED* searches an optimum set of filter *W_k_* coefficients that recover the output signal with the maximum value of kurtosis. For convenience, the filter can be normalized as:

(20) $$\sum_{k=1}^{L}W_{k}^{2}=1$$

**Figure 10:**
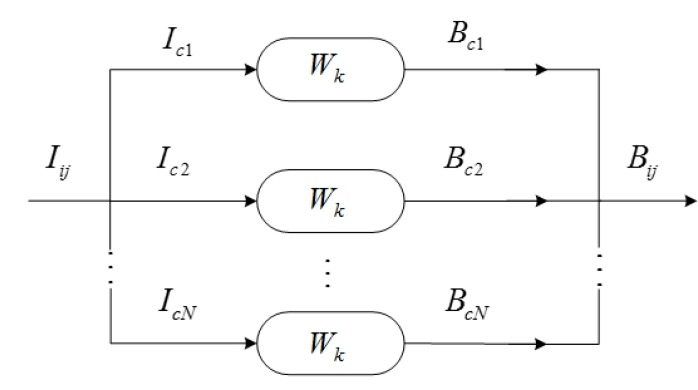
The *MED* model of the multichannel signal.

In accordance with this constraint, it is feasible to conclude that *I_ij_* is converted into *B_ij_*, with its energy preserved and its entropy reduced. The reason can be found through explaining Equation (18) mathematically. In Equation (18), it can be seen that *B_ij_* is obtained by weighting, shifting, overlapping and adding the corresponding components of *I_ij_* (1 < *L* < *N*). (*i*) If *L* = 1, the filter has no impact on the output; (*ii*) if *L* ≥ *N*, the last *L* − *N* + 1 elements has no impact on the output; (*iii*) it is a well-posed estimation problem because there are fewer parameters than those in [42]. Generally, the greater *L* is, the more easily the kurtosis *K* converges to a high value. An appropriate *L* should balance kurtosis, the energy and the computation. Therefore, from the prospect of minimal entropy or maximal kurtosis, the criterion is not the optimal but an acceptable result. Gonzalez suggests that the *L* value should lie between 50% and 100% of the number of elements per segment [38]. In our experiments, *L* is empirically defined as 50% which satisfy the experiment requirements. The deconvolution model (Figure 9) of this problem can be described as Figure 10.

The extremum of Equation (19) is obtained by Equation (21):

(21) $$\frac{\partial O\left(W_{k}\right)}{\partial W_{k}}=0$$

An iteratively converging local-maximum solution can be derived as:

(22) $$\sum_{l=1}^{L}W_{l}\sum_{j=1}^{N}K_{j}U_{j}^{-1}\sum_{i=1}^{M}I_{i-l,j}I_{i-k,j}\text{=}\sum_{j=1}^{N}U_{j}^{-2}\sum_{i=1}^{M}{\left(B_{ij}-\mu_{Bj}\right)}^{3}I_{i-k,j}$$

where:

(23) $$U_{j}=\sum_{i=1}^{M}{\left(B_{ij}-\mu_{Bj}\right)}^{2}$$

(24) $$K_{j}=\frac{\sum_{i=1}^{M}{\left(B_{ij}-\mu_{Bj}\right)}^{4}}{{\left(\sum_{i=1}^{M}{\left(B_{ij}-\mu_{Bj}\right)}^{2}\right)}^{2}}$$

and *W_k_* is iteratively selected. The general procedure is listed in Table 2.

**Table 2:** General Procedure of *MED*.

Step	Algorithm
1	Initializing the adaptive FIR filter, and setting *W_k_* = [11...1...11]/$\sqrt{L}$, *K* = 0.
2	Computing the output signal *B_ij_* according to Equation (11).
3	Inputting *B_ij_* to Equations (22)–(24), *W_k_* is obtained.
4	Inputting *B_ij_* to Equation (19) to compute kurtosis *K* and ΔK.
5	Repeating step 2 and 3 to make sure that a specified number of iterations is achieved and that the change in *K* between iterations is less than a specified small value.

**Figure 11:**
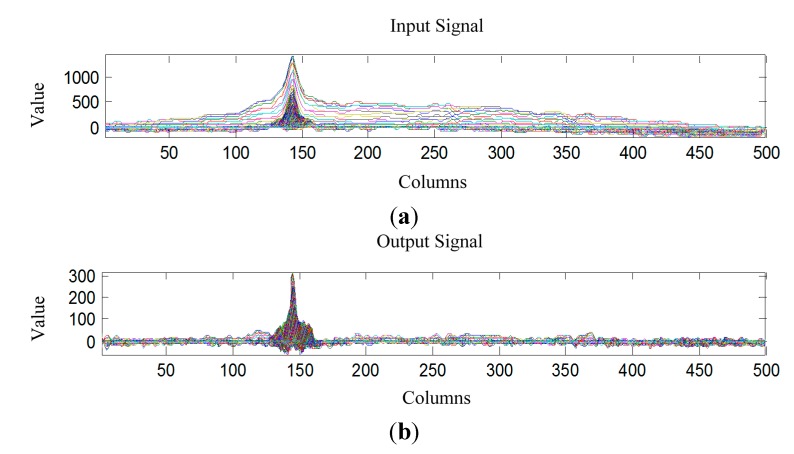
The comparison of energy concentration before and after *MED* processing. (**a**) waveform of the input signal in the columns; (**b**) waveform of the output signal in the columns.

Figure 11 shows the comparison of energy concentration between the input signal and output signal (deconvolved signal by *MED*).

Obviously, after MED processing, SNR is higher and the peaks of laser stripe are much steeper. For the segmentation of the vertical laser stripe, its process is the same as that of the horizontal laser stripe.

### 3.3. Centre Points Localization of Laser Stripe

The comparison between a group of delay in rows (or columns) and the ideal curve should be taken into account. This phase shift can be computed according to 2D convolution theorem. The Equation (25) can be obtained by Equation (18):

(25) $$W_{k}*I_{ij}=\left[\begin{array}{c} B_{ij}\\ Z \end{array}\right]$$

where *Z* is a (*L* − 1) × *N* zeros matrix, and:

(26) $$\Delta i=P_{I}+P_{W},\Delta j=0$$

in which *P_I_* and *P_W_* are the phases of *I_ij_* and *W_k_*, respectively. Δ*i* and Δ*j* are vertical and horizontal displacements from the segmented stripe to the original stripe, respectively. In the same way, the vertical phases and displacements can be calculated. Therefore, to segment the vertical laser stripe in rows has the same process.

**Figure 12:**
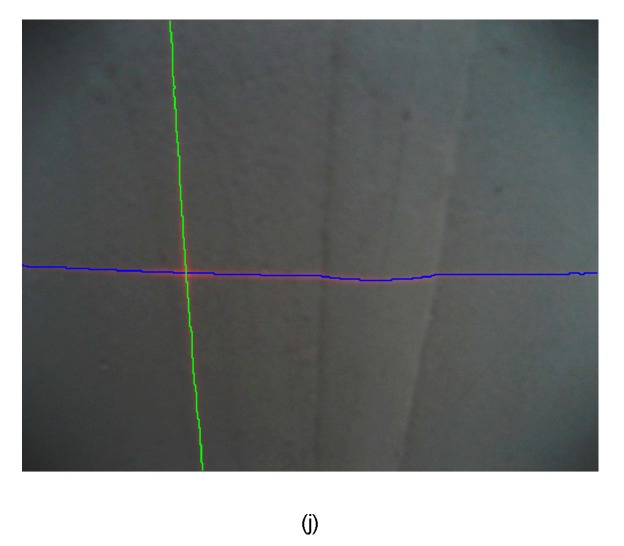
Localization results of the centre points.

Based on the above results of stripe segmentation and illumination restoration, Steger’s method is used to extract the centre points of laser stripes [25]. The method provides good localization results of centre points using Gaussian filtering and edge detection. Figure 12 shows the localization results of the centre points.

## 4. Results and Discussion

Three experiments are conducted, which are the segmentation of the stripe in a weld line image, 3D measurement of the weld line, and tracking the weld line on wall-climbing robot. The proposed sensor and approach are tested in five sets of video data captured from the robot on a wind power tower. The configuration of the CSL sensor is listed in Table 3. These algorithms are implemented in C++ and are tested on an Intel Core i3-2130CPU of 3.4 GHz.

**Table 3:** Configuration of the CSL sensor.

Device	Parameters
Camera	CCD: SONY: 1/4 inch
	Resolution: 640 × 480 pixels
	Pixel size: 5.6 μm × 5.6 μm
	Frame rate: 20 *fps*
	Focal length: 8 mm
	Field of view: 43.7°
Laser projector	Size: ϕ 9 × 23 mm
	Wavelength: 700 nm
	Operating voltage: DC 5 V
	Operating current: 20–50 mA
	Output power: 250 mW
	Fan angle: 60°

### 4.1. CSL Sensor Calibration

**Figure 13:**
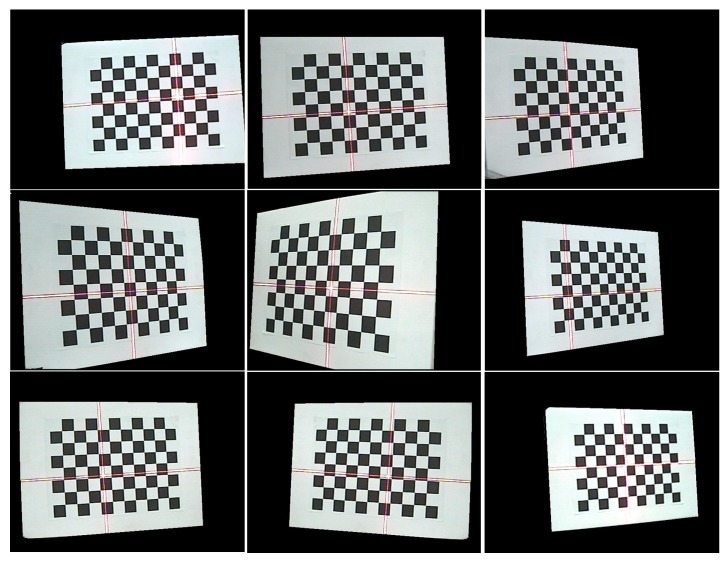
Calibration checkerboard with laser stripe.

A checkerboard is used as a target board of the cameral calibration. On the checkerboard, the side length of each black/white square is equal to 25 mm. There are 10 × 7 corner points on the target board. There are nine captured images of target board in different positions, as shown in Figure 13. The sets of images are used to calibrate camera and calculate equations of laser planes. The coordinates of feature points of laser stripe are extracted by the method in Section 3. Based on the method described in Section 2, the feature points of camera coordinate system are calculated, and then the equations of laser planes are fitted. A plane equation can be obtained by at least three non-collinear feature points. The calibration results of the CSL sensor are listed in Table 4.

**Table 4:** The calibration results of the CSL sensor.

Category	Parameters	Values
Camera intrinsic parameters	(*f_x_*, *f_y_*)	(922.4350, 917.3560)
	(*u*_0_, *v*_0_)	(329.1680, 2705660)
	(*k*_1_, *k*_2_)	(−291.459 × 10^−3^, 157.027 × 10^−3^)
	(*p*_1_, *p*_2_)	(−0.1354 × 10^−3^, −0.2682 × 10^−3^)
Light plane equations	(*a*_1_, *b*_1_, *c*_1_)	(−0.18 × 10^−3^, 1.86 × 10^−3^, 1.39 × 10^−3^)
	(*a*_2_, *b*_2_, *c*_2_)	(−90.11 × 10^−3^, 2.463 × 10^−3^, 8.935 × 10^−3^)
	∠*l*_1_*ol*_2_	89.9981°
Global parameters	*R_cr_*	$\left[\begin{array}{ccc} 0.680 & -0.961 & 0.001\\ 0.732 & 0.704 & -0.027\\ 0.019 & 0.705 & 0.999 \end{array}\right]$
	*T_cr_*	${\left[\begin{array}{ccc} -1.618 & 350.480 & -59.871 \end{array}\right]}^{T}$

It can be seen in Table 5 that the dot product of the coefficients of the two plane equations is equal to *a*_1_*a*_2_ + *b*_1_*b*_2_ + *c*_1_*c*_2_ = 3.322 × 10^−5^, which is close to zero. The measurement accuracy of the CSL is evaluated by comparing standard values and measured values [43]. The standard value is the coordinates of the intersection points of the target board and rays of light between the optical center and feature points, as shown in Figure 3. The measured value is the coordinates of the intersection points of the laser planes and rays of light between optical center and feature point. In a random position, 18 feature points are selected to evaluate measurement accuracy of the system using the laser plane equations.

**Table 5:** Measurement accuracy.

Image Coordinates	Standard Value			Measured Value			Errors of Coordinates		
(u,v)/(pixels)	*x* (mm)	*y* (mm)	*z* (mm)	*x* (mm)	*y* (mm)	*z* (mm)	Δ*x* (mm)	Δ*y* (mm)	Δ*z* (mm)
434.812, 216.242	224.751	−61.644	237.893	224.542	−61.586	237.671	−0.209	0.058	−0.222
521.702, 339.656	208.124	−60.198	231.686	207.856	−60.121	231.388	−0.268	0.077	−0.298
520.861, 304.699	191.494	−58.802	225.479	191.424	−58.781	225.397	−0.070	0.021	−0.082
519.817, 272.006	174.863	−57.407	219.272	174.962	−57.439	219.395	0.099	−0.032	0.123
518.237, 238.050	166.850	−56.695	216.280	166.851	−56.695	216.281	0.001	0	0.001
516.309, 220.555	158.236	−55.971	213.065	158.309	−55.996	213.163	0.073	−0.025	0.098
515.171, 204.063	141.610	−54.515	206.858	141.588	−54.507	206.826	−0.022	0.008	−0.032
512.486, 170.342	124.986	−53.019	200.650	124.763	−52.925	200.291	−0.223	0.094	−0.359
508.894, 138.080	177.332	57.495	216.540	177.262	57.472	216.455	−0.070	−0.023	−0.085
577.181, 225.223	175.097	32.586	216.503	175.014	32.571	216.399	−0.083	−0.015	−0.104
565.821, 224.684	172.689	7.687	216.400	172.695	7.687	216.408	0.006	0	0.008
554.325, 223.663	170.521	−17.226	216.388	170.473	−17.221	216.328	−0.048	0.005	−0.060
539.553, 223.101	168.219	−42.131	216.325	168.197	−42.126	216.297	−0.022	0.005	−0.028
525.946, 222.546	165.957	−67.039	216.278	165.937	−67.031	216.251	−0.02	0.008	−0.027
510.778, 220.525	163.709	−91.947	216.235	163.683	−91.932	216.200	−0.026	0.015	−0.035
494.025, 219.462	161.487	−116.857	216.202	161.441	−116.823	216.14	−0.046	0.034	−0.062
477.062, 218.398	159.212	−141.763	216.150	159.175	−141.730	216.099	−0.037	0.033	−0.051
457.027, 217.322	156.884	-166.667	216.078	156.884	−166.667	216.078	0	0	0
RMS errors (mm)	--	--	--	--	--	--	0.094	0.034	0.120

**Figure 14:**
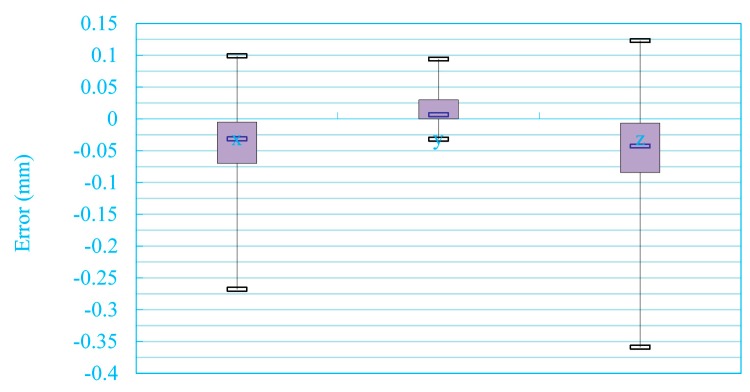
Measurement errors in *x*, *y* and *z* directions.

**Figure 15:**
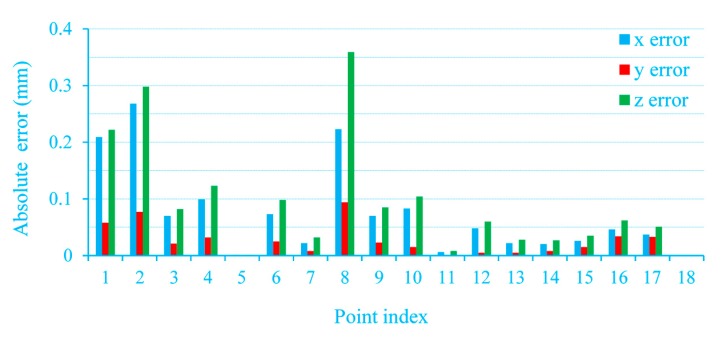
The absolute errors in feature points.

Table 5 lists the measurement accuracy evaluation data. There are Root-Mean-Square (RMS) errors of (0.094, 0.034, 0.120) in the three directions. The measurement errors are shown in Figure 14 and Figure 15.

### 4.2. Accuracy and Speed of Stripe Segmentation

This paper quantitatively contrasts the performance of the proposed approach (*MED*), Centre of mass (*CM*), Linear approximation (*LA*), quadratic approximation (*QA*), and Akima splines approximation (*AA*). The different monochromatic value spaces (*R* or *R-G*), absolute errors and running time are computed, respectively.

**Figure 16:**
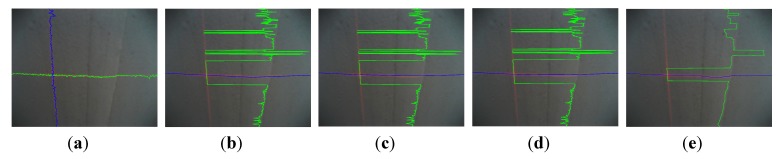

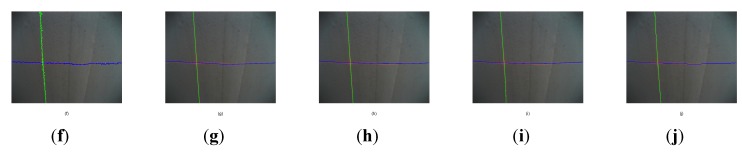
The segmentation result of the above methods in *R* component space and *R-G* space: (**a**–**e**) and (**f**–**j**) are results of *CM*, *LA*, *QA*, *AA* and *MED* in *R* and *R-G* respectively.

**Figure 17:**
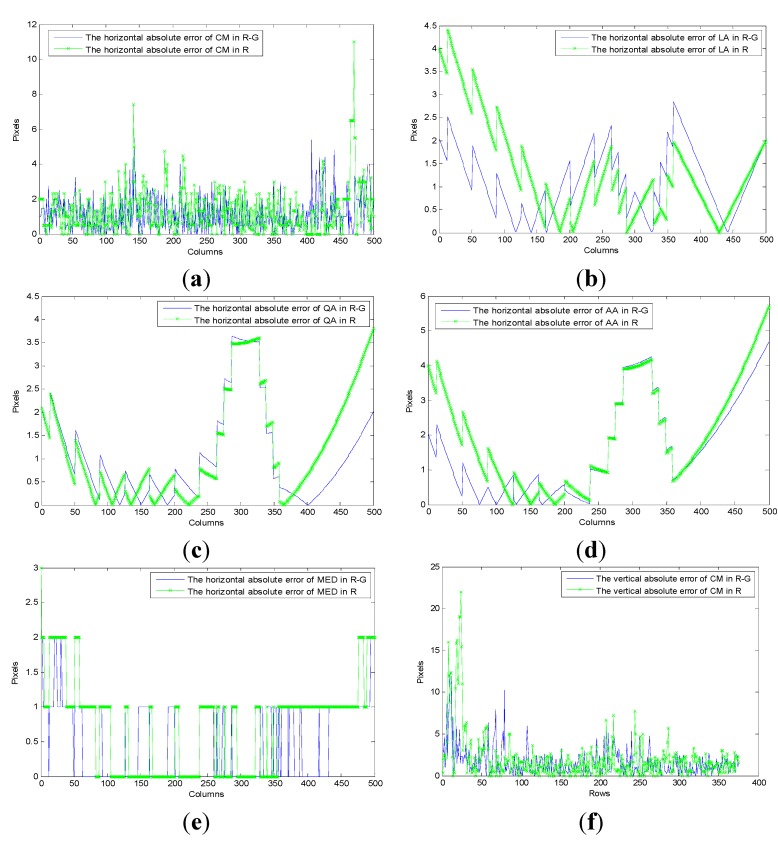

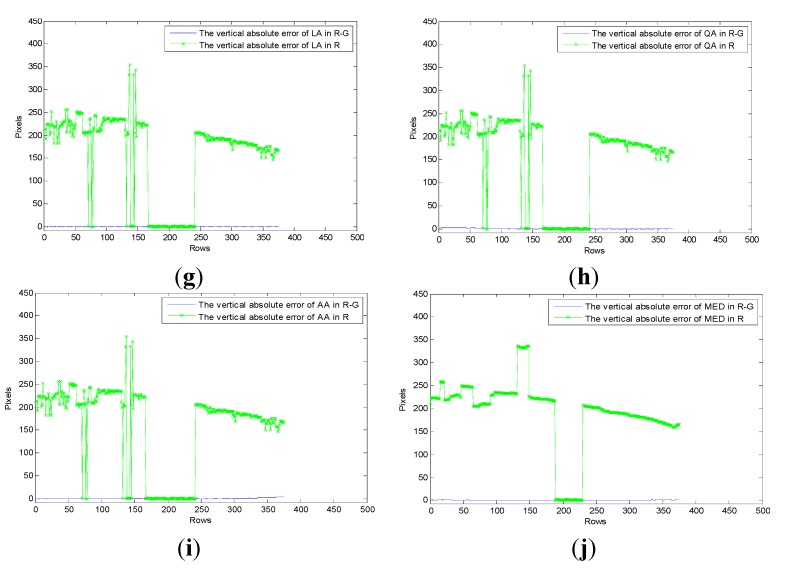
The errors of the above methods in *R* and *R-G*: (**a**–**e**) are errors of the horizontal stripe using *CM*, *LA*, *QA*, *AA* and *MED* in *R* and *R-G* respectively; (**f**–**j**) are errors of the vertical stripe using *CM*, *LA*, *QA*, *AA* and *MED* in *R* and *R-G* respectively.

In the test images, the segmentation quality of the comparison algorithms and the proposed algorithm are shown in Table 6, Figure 16 and Figure 17. The runtime per image is the mean value obtained by the processing of 1000 images. As a whole, it can be seen from the following table and figures that (*i*) the component *R-G* is better than *R* in all methods; (*ii*) the method of segmenting laser stripe based on *MED* is more robust, accurate, and fast than other approaches. It is worth noting that the data of vertical laser stripe obtained in *R* is so tough that the time it consumed is too long to afford in practice. Taking this factor into account, it is unfeasible to use *LA*, *QA*, *AA* and *MED* to fit the vertical laser stripe.

**Table 6:** Laser Stripe Segmentation Performance.

Laser Stripe	Color Space		Method	*CM*	*LA*	*QA*	*AA*	*MED*
		Index						
Horizontal Laser stripe	*R*	Average error (mm)		0.432	0.667	0.271	0.311	0.231
		Running time (ms)		18.3	320.2	168.1	130.3	22.3
	*R-G*	Average error (mm)		0.330	0.416	0.267	0.291	0.231
		Running time (ms)		17.9	314.0	167.6	196.6	20.9
Vertical Laser stripe	*R*	Average error (mm)		1.001	66.710	73.334	70.350	71.050
		Running time (ms)		18.8	^∞^	^∞^	^∞^	20.6
	*R-G*	Average error (mm)		0.700	0.431	0.295	0.327	0.235
		Running time (ms)		17.6	120.2	147.2	166.6	19.9

“^∞^”means that the time is too long to afford in practice, *num* = 500.

Nonetheless, the error of *MED* overall is smaller than the former. Furthermore, they are smoother than the fitting data obtained by *LA*, *QA*, and *AA* in *R-G*. This phenomenon is determined by the more flat distribution of the former error, though most of its elements are larger and they have similar offsets relative to the true data. Another contribution of the proposed method is that the errors of the vertical stripe by *MED* are only larger than those of *LA* in *R-G* space, which results from different reasons. Firstly, the vertical laser stripe is fitted by linear approximation of *LA*, and it is a straight line in the image. Secondly, since only a few points with a lager error value have useful information, the breakpoints detection is absent in *MED*, which makes the method more effective and robust than *LA*. And the vertical laser stripe by *MED* is smoother than *LA*. Therefore, the performance of *MED* is better.

**Figure 18:**
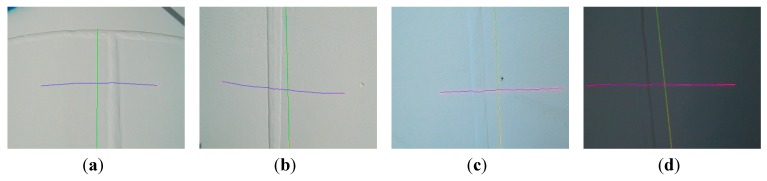
Laser stripe segmentation results with different illumination interferences. (**a**,**b**) Cross and straight weld line in the sunshine; (**c**) The weld line with reflections surface; (**d**) The weld line in the shadow.

Figure 18 shows the laser stripes extraction results in four frames captured in the wild environment. Despite high illumination and varying intensity of light in the frames, the algorithm exhibits good performance. All the calculations are based on the full image. It can be seen that the speed of *MED* is fast enough to be applied in the real environments. If the region of interest is selected, the running speed will become faster.

### 4.3. Weld Line Detection and Tracking of Wall Climbing Robot

**Figure 19:**
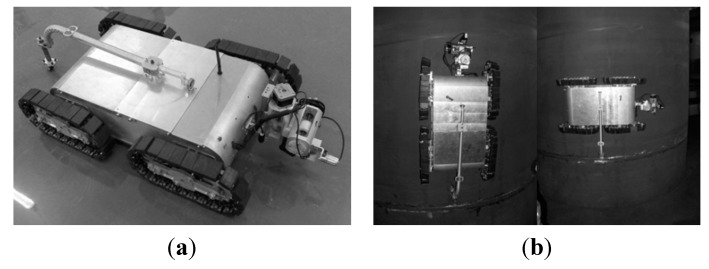
Wall climbing robot prototype. (**a**) Robot with the CSL device; (**b**) Robot working in vertical and horizontal direction.

**Figure 20:**
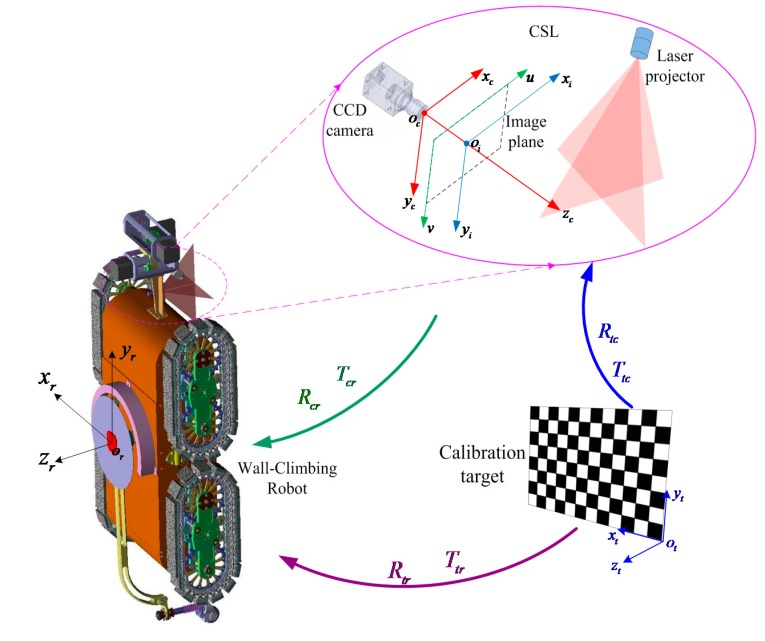
The conversion of the camera coordinate system, the robot coordinate system and the calibration target coordinate system.

In order to verify the feasibility of the CSL sensor, the calibrated sensor is tested on a wind power tower by a wall climbing robot platform, as shown in Figure 19. Figure 20 describes the conversion of the camera coordinate system, the robot coordinate system and the calibration target coordinate system. In which (*R_c__r_*, *T_c__r_*), (*R_t__r_*, *T_t__r_*) and (*R_t__c_*, *T_t__c_*) denote rotation matrixes and translational matrixes between three coordinate systems, respectively. The location of the weld line is detected by the method in [27]. According to the detection results, the robot can follow the weld line and perform NDT.

**Figure 21:**
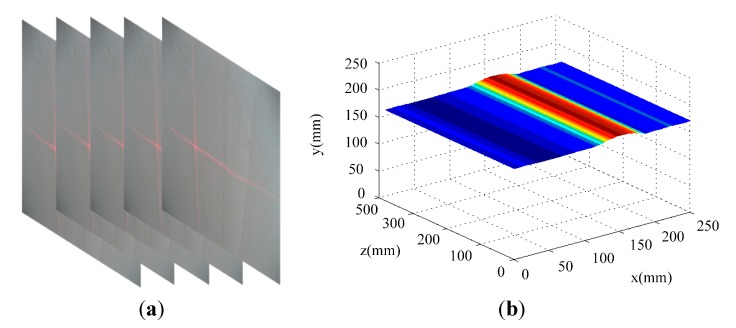

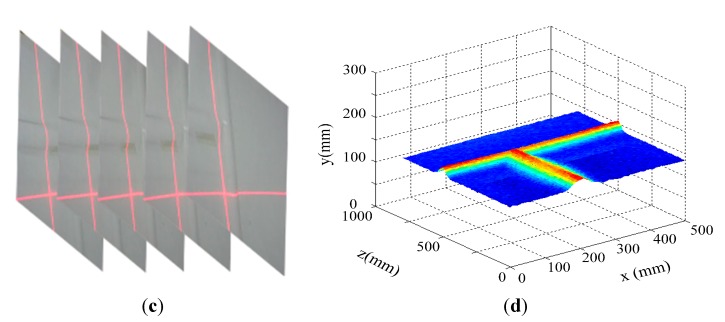
Measurement and 3D coordinates of straight and cross weld lines. (**a**) Measure straight weld line; (**b**) 3D coordinates of the straight weld line; (**c**) Measure cross weld line; (**d**) 3D coordinates of the cross weld line.

In the paper, the measured object is the weld line of a wind power tower. The tower is a bulky cone with a height of about 30–50 m and a diameter of about 2.2–4 m. Therefore, the structured light sensor may not reconstruct 3D information of the whole tower. We just reconstruct the local 3D information around the weld line. Figure 21 shows the detection and measurement experiments in the wild environment. The robot coordinate system is supposed to be the global coordinate system. In practice, the speed of the wall climbing robot is about 30 mm/s (about 1.5 mm/frame given a video frame rate of 20 fps). For the captured image with 640 × 480 pixels, the difference between two successive frames is about 5~6 row pixels. Therefore, the percent overlap is about 99%. Under the circumstances of high illumination and excessive noise, the robustness of the sensor stands out. Figure 21b shows the extracted laser stripe on the weld lines. Figure 21c illustrates the detection results of the T-intersection weld lines and Figure 21d shows the measurement results of the T-intersection weld lines. Both vertical and horizontal weld lines appear in the video sequence. It can be seen that the CSL sensor can detect and measure both vertical and horizontal weld lines when the robot moves close to the intersection part of the two weld lines. From the image space to the object space the coordinate transforming relation is:

(27) $${\left[x_{r},y_{r},z_{r}\right]}^{T}=R_{cr}K^{-1}{\left[u,v,\;1\right]}^{T}+T_{cr}$$

**Figure 22:**
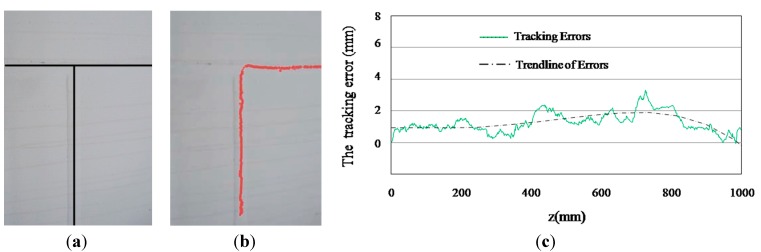
Weld line tracking results of the wall climbing robot. (**a**) Central lines of two cross weld lines (ground-truth); (**b**) Motion trail of the robot; (**c**) The tracking errors.

When the robot moves along the straight weld lines, the measured weld line locations can be used directly for navigation. When the robot moves close to T-intersection weld lines, we can get two convex arcs on the laser stripes, and the vertical and horizontal locations of the weld lines can be extracted simultaneously using the CSL sensor. In Figure 22, we show the navigation and motion trajectories of the robot at the T-intersection of the weld lines. The average tracking error is less than 2 mm to ideal central line.

The camera does not have any optical filter, so that the segmentation process becomes more adaptive and lower cost compared with other ways used in the industry. Although a high noise level exists in low-quality images, the sensor and the proposed algorithm are robust enough to segment the laser stripe and detect weld lines accurately.

## 5. Conclusions

This paper proposes a structured light sensor with cross stripes and a segmentation algorithm of the stripe for vision navigation of a wall climbing robot in outdoor environments. The simple but effective cross structured light device provides an example for weld line measurement, while we provide a general methodology based on MED for a kind of measurement problems in industrial applications. The proposed algorithm chooses an adaptive monochromatic value space for preprocessing, and then recovers the laser stripe after the deconvolution process.

To process a color image with 640 × 480 pixels resolution, the average running time is about 20 ms. In cameral calibration, RMS of measurement errors is within 0.120 mm. In the field experiments, the absolute measurement error is less than 0.3 mm. In the robot navigation experiments, the average tracking error is less than 2 mm. The results of the experiments demonstrate that the designed sensor and the proposed algorithm have high accuracy, robustness and efficiency for measurement and navigation.

## References

[1] Silberman N., Rob F. Indoor scene segmentation using a structured light sensor. Proceedings of the IEEE International Conference on Computer Vision Workshops.

[2] Park J.B., Lee S.H., Lee J. (2009). Precise 3D lug pose detection sensor for automatic robot welding using a structured-light vision system. Sensors.

[3] Huang W., Radovan K. (2011). A laser-based vision system for weld quality inspection. Sensors.

[4] Zhao X., Liu H., Yu Y., Xu X., Hu W., Li M., Ou J. (2015). Bridge Displacement Monitoring Method Based on Laser Projection Sensing Technology. Sensors.

[5] Usamentiaga R., Molleda J., Garcia D.F. (2014). Structured-Light Sensor Using Two Laser Stripes for 3D Reconstruction without Vibrations. Sensors.

[6] Barone S., Alessandro P., Armando V.R. (2012). 3D Reconstruction and Restoration Monitoring of Sculptural Artworks by a Multi-Sensor Framework. Sensors.

[7] Zhan D., Yu L., Xiao J., Chen T. (2015). Multi-Camera and Structured-Light Vision System (MSVS) for Dynamic High-Accuracy 3D Measurements of Railway Tunnels. Sensors.

[8] Bieri L.S., Jacques J. (2004). Three-dimensional vision using structured light applied to quality control in production line. Proc. SPIE.

[9] Usamentiaga R., Molleda J., García D.F., Bulnes F.G. Machine vision system for flatness control feedback. Proceedings of the IEEE International Conference on Machine Vision.

[10] Appia V., Pedro G. (2014). Comparison of fixed-pattern and multiple-pattern structured light imaging systems. Proc. SPIE.

[11] Gupta M., Qi Y., Nayar S.K. Structured light in sunlight. Proceedings of the IEEE International Conference on Computer Vision.

[12] O’TOOLE M., John M., Kutulakos K.N. 3D shape and indirect appearance by structured light transport. Proceedings of the 2014 IEEE Conference on Computer Vision and Pattern Recognition.

[13] Liu D., Cheng X., Yang Y.-H. Frequency-Based 3D Reconstruction of Transparent and Specular Objects. Proceedings of the 2014 IEEE Conference on Computer Vision and Pattern Recognition.

[14] Molleda J., Usamentiaga R., García D.F., Bulnes F.G. (2010). Real-time flatness inspection of rolled products based on optical laser triangulation and three-dimensional surface reconstruction. J. Electron. Imaging.

[15] Usamentiaga R., Molleda J., Garcia D.F., Bulnes F.G. (2014). Removing vibrations in 3D reconstruction using multiple laser stripes. Opt. Lasers Eng..

[16] Fisher R.B., Naidu D.K. (1996). A comparison of algorithms for subpixel peak detection. Image Technology.

[17] Haug K., Pritschow G. Robust laser-stripe sensor for automated weld-seam-tracking in the shipbuilding industry. Proceedings of the IEEE Annual Conference of the Industrial Electronics Society.

[18] Strobl K.H., Sepp W., Wahl E., Bodenmuller T., Suppa M., Seara J.F., Hirzinger G. The DLR multisensory hand-guided device: The laser stripe profiler. Proceedings of the 2004 IEEE International Conference on Robotics and Automation.

[19] Li Y., Li Y.F., Wang Q.L., Xu D., Tan M. (2010). Measurement and defect detection of the weld bead based on online vision inspection. IEEE Trans. Instrum. Meas..

[20] Molleda J., Usamentiaga R., Garcia D.F., Bulnes F.G., Ema L. (2011). Shape measurement of steel strips using a laser-based three-dimensional reconstruction technique. IEEE Trans. Ind. Appl..

[21] Usamentiaga R., Molleda J., García D.F. (2012). Fast and robust laser stripe extraction for 3D reconstruction in industrial environments. Mach. Vis. Appl..

[22] Ofner R., O’Leary P., Leitner M. (1999). A collection of algorithms for the determination of construction points in the measurement of 3D geometries via light-sectioning. Workshop on European Scientific and Industrial Collaboration: Advanced Technologies in Manufacturing.

[23] Forest J., Salvi J., Cabruja E., Pous C. Laser stripe peak detector for 3D scanners. A FIR filter approach. Proceedings of the IEEE International Conference on Pattern Recognition.

[24] Schnee J., Futterlieb J. (2011). Laser line segmentation with dynamic line models. Computer Analysis of Images and Patterns.

[25] Steger C. (1998). An unbiased detector of curvilinear structures. IEEE Trans. Pattern Anal. Mach. Intell..

[26] Xu D., Wang L., Tu Z., Tan M. (2005). Hybrid visual servoing control for robotic arc welding based on structured light vision. Acta. Autom. Sin..

[27] Zhang L., Ye Q., Yang W., Jiao J. (2014). Weld line detection and tracking via spatial-temporal cascaded hidden Markov models and cross structured light. IEEE Trans. Instrum. Meas..

[28] Sturm P., Ramalingam S., Tardif J.P., Gasparini S., Barreto J. (2011). Camera models and fundamental concepts used in geometric computer vision. Found. Trends Comp. Graph. Vis..

[29] Tsai R.Y. (1987). A versatile camera calibration technique for high-accuracy 3D machine vision metrology using off-the-shelf TV cameras and lenses. IEEE J. Robot. Autom..

[30] Medioni G., Kang S.B. (2004). Emerging Topics in Computer Vision.

[31] Zhang Z. (2000). A flexible new technique for camera calibration. IEEE Trans. Pattern Anal. Mach. Intell..

[32] Moré J.J. (1978). The Levenberg-Marquardt algorithm: Implementation and theory. Numerical Analysis.

[33] Wiggins R.A. (1978). Minimum entropy deconvolution. Geoexploration.

[34] McDonald G.L., Zhao Q., Zuo M.J. (2012). Maximum correlated Kurtosis deconvolution and application on gear tooth chip fault detection. Mech. Syst. Signal Process..

[35] Collins R.T., Liu Y., Leordeanu M. (2005). Online selection of discriminative tracking features. IEEE Trans. Pattern Anal. Mach. Intell..

[36] Ohta Y.I., Kanade T., Sakai T. (1980). Color information for region segmentation. Comput. Graph.Image Process..

[37] Bronstein M.M., Bronstein A.M., Zibulevsky M., Zeevi Y.Y. (2005). Blind deconvolution of images using optimal sparse representations. IEEE Trans. Image Process..

[38] González G., Badra R.E., Medina R., Regidor J. (1995). Period estimation using minimum entropy deconvolution (*MED*). Signal Process..

[39] Sawalhi N., Randall R.B., Endo H. (2007). The enhancement of fault detection and diagnosis in rolling element bearings using minimum entropy deconvolution combined with spectral kurtosis. Mech. Syst. Signal Process..

[40] Nandi A.K., Mämpel D., Roscher B. (1997). Blind deconvolution of ultrasonic signals in nondestructive testing applications. IEEE Trans. Signal Process..

[41] Boumahdi M., Lacoume J.L. Blind identification using the Kurtosis: Results of field data processing. Proceedings of the IEEE International Conference on Acoustics, Speech, and Signal Processing.

[42] Donoho D. (1981). On minimum entropy deconvolution. Applied Time Series Analysis II.

[43] Zhou F., Peng B., Cui Y., Wang Y., Tan H. (2013). A novel laser vision sensor for omnidirectional 3D measurement. Opt. Laser Technol..

